# Customizable large-scale HPLC fraction collection using low-cost 3D printing

**DOI:** 10.1016/j.ohx.2024.e00612

**Published:** 2024-12-10

**Authors:** William J. Crandall, Marco Caputo, Lewis Marquez, Zachery R. Jarrell, Cassandra L. Quave

**Affiliations:** a Molecular and Systems Pharmacology Program, Emory University, Atlanta, GA, USA; b Department of Dermatology, Emory University School of Medicine, Atlanta, GA, USA; c Jones Center at Ichauway, Newton, GA, USA; d Division of Pulmonary, Allergy, Critical Care, and Sleep Medicine, Emory University, Atlanta, GA 30322, USA

**Keywords:** HPLC, Fraction Collector, Low-Cost, 3D Printing, Sample Collection

## Abstract

High-performance liquid chromatography (HPLC) is an invaluable technique that has been used for many decades for the separation of various molecules. The reproducible collection of eluates from these systems has been significantly improved via its automation by fraction collection systems. Current commercially available fraction collectors are not easily customizable, incompatible with other platforms, and come with a large cost barrier making them inaccessible to many researchers. Here we present the efficient construction of a low-cost customizable fraction collector that can easily be paired to any HPLC system. Notably, it supports significantly larger volumes for collection than commercial alternatives. Using a hobbyist-grade three-dimensional (3D) printer (Creality Ender 3 Pro) and aluminum extrusions, the fraction collector can be built for less than $280 USD. An additional graphical user interface (GUI) enables simple programming of the collection methods, requiring no coding experience to operate the collector. The presented fraction collector can be highly customized and use collection vessels as large as 470 mL (80x), facilitating repeated collection at a preparatory scale. The use of this platform will increase the reproducibility of scalable and iterative fraction collection methods while removing the cost barrier and allowing for a high degree of customizability.

## Graphical abstract

Using a 3D Printer, additional hardware, and a graphical user interface (GUI) written in Python, an Ender 3 Pro can be transformed to a large scale fraction collector (73 cm x 70 cm x 31 cm).

**Figure:**
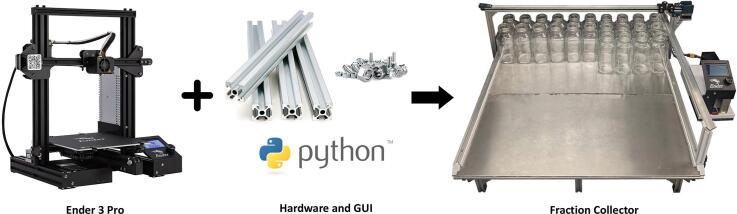

**Specifications table**.

**Table:** 

Hardware name	Customizable Large-Scale Fraction Collector
Subject area	• Chemistry and biochemistry
Hardware type	• Sample handling and preparation
Closest commercial analog	• GE AKTA Frac-950 Fraction Collector. (Free standing time/volume-based fraction collector capable of multiple collection vessels). (GE AKTA Frac-950 Fraction Collector | Marshall Scientific).
Open source license	GNU General Public License
Cost of hardware	$280 USD
Source file repository	https://doi.org/10.5281/zenodo.14026247

## 1 Hardware in context

High-Performance Liquid Chromatography (HPLC) has been used for many decades for the separation of numerous types of molecules [1], [2], [3], [4], [5], [6]. Increased flow rates coupled with larger columns allow for the efficient isolation of sufficient quantities of molecules from complex samples, a technique termed “preparative HPLC”. It is particularly powerful for the isolation of small molecule natural products, given the enormous chemical complexity of the starting material. Manual collection of the eluate from HPLC systems, however, is a time-consuming process for researchers and can be prohibitive towards reproducibility, thus automated fraction collection is a desired, if not necessary, addition to many LC systems.

The cost, compatibility, and customizability of these systems present a large barrier to access to researchers and limit their usefulness. This is evident in the field of natural product isolation, where individual compounds can vary greatly in the percent composition of the starting extract. A straightforward approach to obtaining larger amounts of isolated material is by scaling the isolation method. Current commercially available fraction collectors are expensive, allow for collection in only a few types of bottles, and are not optimized for this purpose. This open-source fraction collector is low-cost, easy to program, and compatible with a variety of bottle types, capable of collecting up to a total of 37 L of solvent. The closest commercial analogue is a time/volume-based fraction collector from General Electric, Boston, MA, USA (AKTA Frac-950) which is priced at around $3,500 USD. Similar equipment can be found used for around $1000 USD. It provides similar functionality, yet is over 12 times more expensive, not fully customizable, and has roughly 5–10 % of the total volume capacity as this open-source model.

The cost or lack of customizability for liquid fraction collectors has been an issue which multiple open source and DIY fraction collectors have been developed to solve. Some of which are more niche applications in either microliter small volume multi-channel applications for protein/peptide fractionation and or spotting for MALDI (matrix-assisted laser desorption ionization) Mass Spectrometry applications [7], [8]. Others require conversion from much more expensive equipment [9], are limited to a single collection vessel size too small for application in isolation [10], or are only one-dimensional parallel collectors and thus limited to the number of tubes held in a single row [11]. Carvalho et al. [12] have used a 3D printer as a fraction collector, using the heated bed of the printer and an aluminum sample block to rapidly evaporate small volumes of eluates for compound specific isotope analysis. However, it is more difficult to program a method and limited in collection vessel size and therefore, its application to preparatory scale HPLC. Medina et al. [13] have developed an open-source smart fraction collector that switches peaks based on signals from the detector. However, it is limited to isocratic systems due to a threshold above baseline signal being needed for peak switching. A more complex algorithm for determining peaks is required for gradient elution, due to changing baseline signal. Isocratic systems are inadequate for the complex sample separation outlined in our use case. While most HPLCs have 5 V signal outputs, implementation of this signal greatly increases build complexity through the wiring and programming of an Arduino board, in addition to total cost. The simplicity that time-based fraction collection affords benefits the pairing of such a device to multiple types of liquid-based chromatographic systems such as flash chromatography systems like the Biotage Isolera One Flash (Biotage Uppsala, Sweden) or Teledyne ISCO CombiFlash (Thousand Oaks, USA) in addition to HPLCs.

Our group has previously designed a fraction collector for high throughput applications of large volume preparative HPLC [14]. In terms of ease of use, a method for the fraction collection can easily be programmed by simply typing in the duration of collection for each fraction. This is a significant improvement over our previous model, which required the method to be programmed in LEGOMINDSTORMS© block-based programming. This made creating methods more time consuming. Additionally, this fraction collector is much more robust in its construction and was constructed at a lower cost. No coding, soldering, or advanced wiring of control boards is required in the construction or use the fraction collector in its current configuration, allowing ease of use for other researchers. Implementation of a fraction collector capable of holding bottles with large volumes (∼470 mL) would complement the high flow rate (5 – 50 mL min^−1^) of preparatory HPLCs, allowing for iterative collections of eluates without constantly replacing collection vessels or combining fractions. The premise of this fraction collector is that a low-cost 3D printer can be purchased and then transformed into the fraction collector itself. With the necessary firmware and software to replicate this build, it can be built solely by 3D printing parts and assembling the collector (Table 1).

**Table 1:** A table containing all part numbers, quantities, and descriptions. Parts are referred to as P1-P34 to distinguish them from the 3D-printed parts (named ‘STL’ prefix). Parts in bold on the parts list are salvaged from the 3D Printer.

**Part #**	**Quantity**	**Description**
P1	2	730 mm v-slot 20x20 linear rail (front and back)
P2	3	70 mm v-slot 20x20 linear rail (front legs)
P3	10	V – slot cast corner bracket 2028 (larger)
P4	4	V – slot cast corner bracket 2020 (smaller)
P5	3	GT2 dual-bearing aluminum idle pulley (6 mm belt 5 mm bore)
P6	2	Precision shim (1 x 5.1 x 8 mm)
P7	2	Aluminum spacer (6 mm)
P8	2	M5 nut
P9	3	M5 x 25 screw
P10	44	M5 x 8 button head screw
P11	53	M5 – sliding t-nut
P12	3	90 mm v-slot 20x20 linear rail (back legs)
P13	2	660 mm v-slot 20x40 linear rail (sides)
P14	1	660 mm v-slot 20x20 linear rail (middle)
P15	4	Makerlink 90 hidden t-nut
P16	2	240 mm v-slot 20x20 linear rail
**P17**	**2**	**Adjustable roller (hex)**
**P18**	**4**	**Roller**
**P19**	**1**	**Y-axis motor**
**P20**	**1**	**Motor mount**
P21	1	Smooth rod (3/16 in. x 36 in. cut to length)
P22	1	GT2 – 2 M 20-tooth pulley
P23	3	Belt (GT2 – pitch 2 mm, 6 mm width, 5 m)
**P24**	**2**	**Bearing**
P25	6	Belt clamp/ zip tie
P26	2	M4 x 4 set screw
**P27**	**1**	**X-axis motor**
**P28**	**1**	**X-axis limit switch**
**P29**	**1**	**Roller plate**
P30	1	820 mm v-slot 20x20 linear rail
**P31**	**4**	**M3 x 40 screw**
P32	1	Waste channel (90° Aluminum angle stock 3/4 in. x 1/8 in. x 48 in. cut to length)
P33	1	Base platform (14 gauge / 2 mm thick, ∼28 in. x ∼ 27 in.)
**P34**	**1**	**Y-axis limit switch**
STL1	2	Bearing casing
STL2	2	T-plate roller mount
STL3	1	Symmetric pulley
STL4	1	X-axis motor plate
STL5	1	X-axis motor and switch mount
STL6	1	X-axis tensioner
STL7	1	Front support
STL8	1	Rear support
STL9	1	Front angle cover (left)
STL10	1	Front angle cover (right)
STL11	1	Support mounting plate
STL12	2	Channel tilt mount
STL13	12	Platform edge bumper
STL14	4	Corner bumper
STL15	1	Chain lock
STL16	40	Chain link
STL17	1	Holder base
STL18	1	Holder top

## 2 Hardware description

The proposed Fraction Collector is large scale, easy to implement, highly customizable, and low cost. Coupled with an LC system, it would be most useful to researchers interested in the isolation of a variety of molecules across fields:

• Natural Products Chemistry – Isolation of small molecules from complex mixtures
• Organic Chemistry – Purification of synthesized products
• Biochemistry – Isolation of biomolecules or metabolites
• Drug Discovery – Purification or isolation of small molecules of interest

The faction collector was developed in our laboratory for the isolation of natural products, small molecules derived from natural sources, however, it can be paired to any LC system and customized for uses requiring automated sample collection. Natural products typically require extensive isolation efforts to obtain novel structures. They are not typically the most abundant compound present in the starting material, making isolating sufficient quantities for structure elucidation quite challenging. For example, individual compounds can vary greatly in the percent composition of the starting extract and can often be found in quantities of 0.68 – 1.2 % [15] or less. Measuring the amount isolated by the weight of dried plant material, this amount can be even smaller (0.0019 %) [16]. Repeated, automated fraction collection on any liquid chromatographic instrument would benefit from the ability to reliably collect large volumes of eluate.

The ability to fabricate parts in a laboratory by way of 3D printing has enabled many advancements in science, from useful equipment, assay plates, sample holders, and sensors, to developing heart valves using biocompatible materials or even cells [17], [18]. Three-dimensional printing is an additive manufacturing technique that first became a commercial enterprise in 1986 [19] with the patenting of stereolithography and in 1989 with the patenting of FDM (fusion deposition modeling). However, it was not until 2009 when the U.S. patent for FDM expired that cheap, hobbyist-grade printers became available. Today these hobbyist-grade printers have increased in quality and affordability, to the point where a capable printer can be purchased for as little as $100 – $200 USD (as of 2024). Their implementation in the laboratory setting has become increasingly common and allows for the creation of customized laboratory equipment. The 3D printer used in the construction of this fraction collector was an Ender 3 pro (Creality, USA). This printer was purchased for $99 USD, justifying the salvaging of parts to complete the fraction collector build. All parts used from the 3D printer were recorded if replication of the fraction collector is desired without the disassembly of the 3D printer, however, this is not what was intended. Costs from outsourcing the 3D prints would have to be considered. Additionally, we found that individual purchases of all the required pieces salvaged from the printer to assemble the fraction collector would be less cost-effective (roughly $142 USD at the time of building the collector not considering additional costs for pieces of mounting hardware).

## 3 Design files summary

**Table:** 

**Design file name**	**File type**	**Open-source license**	**Location of the file**
STL1	.stl (stereolithography)	*GNU General Public License v3.0*	https://doi.org/10.5281/zenodo.14026247
STL2	.stl (stereolithography)	*GNU General Public License v3.0*	https://doi.org/10.5281/zenodo.14026247
STL3	.stl (stereolithography)	*GNU General Public License v3.0*	https://doi.org/10.5281/zenodo.14026247
STL4	.stl (stereolithography)	*GNU General Public License v3.0*	https://doi.org/10.5281/zenodo.14026247
STL5	.stl (stereolithography)	*GNU General Public License v3.0*	https://doi.org/10.5281/zenodo.14026247
STL6	.stl (stereolithography)	*GNU General Public License v3.0*	https://doi.org/10.5281/zenodo.14026247
STL7	.stl (stereolithography)	*GNU General Public License v3.0*	https://doi.org/10.5281/zenodo.14026247
STL8	.stl (stereolithography)	*GNU General Public License v3.0*	https://doi.org/10.5281/zenodo.14026247
STL9	.stl (stereolithography)	*GNU General Public License v3.0*	https://doi.org/10.5281/zenodo.14026247
STL10	.stl (stereolithography)	*GNU General Public License v3.0*	https://doi.org/10.5281/zenodo.14026247
STL11	.stl (stereolithography)	*GNU General Public License v3.0*	https://doi.org/10.5281/zenodo.14026247
STL12	.stl (stereolithography)	*GNU General Public License v3.0*	https://doi.org/10.5281/zenodo.14026247
STL13	.stl (stereolithography)	*GNU General Public License v3.0*	https://doi.org/10.5281/zenodo.14026247
STL14	.stl (stereolithography)	*GNU General Public License v3.0*	https://doi.org/10.5281/zenodo.14026247
STL15	.stl (stereolithography)	*GNU General Public License v3.0*	https://doi.org/10.5281/zenodo.14026247
STL16	.stl (stereolithography)	*GNU General Public License v3.0*	https://doi.org/10.5281/zenodo.14026247
STL17	.stl (stereolithography)	*GNU General Public License v3.0*	https://doi.org/10.5281/zenodo.14026247
STL18	.stl (stereolithography)	*GNU General Public License v3.0*	https://doi.org/10.5281/zenodo.14026247

A description of design files and a more detailed parts list are outlined in the build instructions section.

## 4 Bill of materials summary

The detailed bill of materials can be found in the online file repository at (https://doi.org/10.5281/zenodo.14026247).

## 5 Build instructions

The frame of the fraction collector is made from aluminum extrusions of v-slot linear rails fastened with various brackets purchased either from Bulk Man 3D (BULK-MAN 3D – Online Store (bulkman3d.com)) or Open Build Parts Store (OpenBuilds Part Store) depending on availability or which supplier could provide the required parts for a lower cost. Parts P21, P32 were acquired from local hardware stores in the United States such as Home Depot or Ace Hardware, and P33 can be acquired from an online store and cut to custom dimensions (OnlineMetals.com). Listed below are all the additional parts to purchase for the frame construction and 3D printed part.stl files, which can be found in the Zenodo repository. Additional photographs are also available for reference.

Risks are present when assembling aluminum extrusions, as the edges and corners can be quite sharp. Printing in a well-ventilated room minimizes the risk of inhaling small plastic contaminates. Loose clothing and hair should be avoided when operating the fraction collector.

The assembly instruction shows the location of each part and its relative dimensions. Steps in which a new part is introduced provide details as to which hardware is required for assembly. Duplicate parts in each step are identified once unless ambiguity arises but are recorded properly in the parts list.

**Fig. 1:**
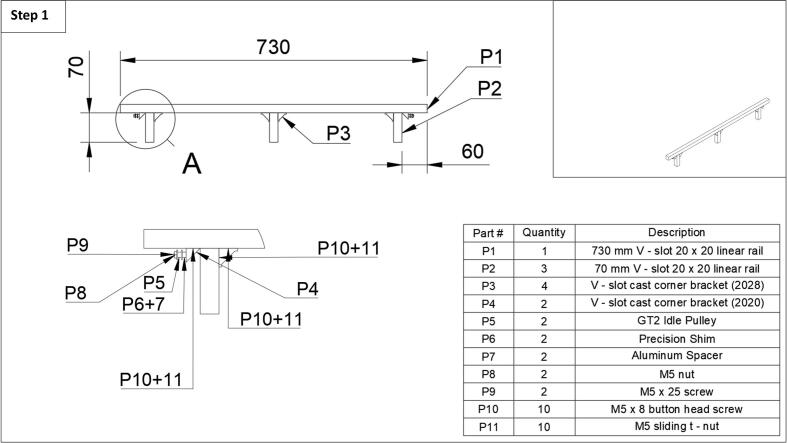
Step 1 of the fraction collector assembly guide.

Step 1: First, the middle leg (P2), should be assembled, as the t-nuts must be slid down the length of the front 730 mm long, 20x20 mm linear rail (P1). Using two t-nuts (P11) and two M5 button head screws (P10), attach the larger cast corner bracket 2028 (P3) to one of the front leg pieces (P2). Next, slide two t-nuts (P11) down each side of the front rail (P1) to the middle, and using two more button-head screws (P10), complete the attachment of the middle leg by screwing in the corner bracket. The same approach can be taken for the outermost legs, this time, however, only using one corner caster 2028 (P3) bracket for each leg (P2) as shown in Fig. 1. They should be positioned roughly 60 mm in from the edge of the rail to give space for the idle pulleys and belts. Next, the pulley assembly can be attached to a smaller corner caster bracket 2020 (P4). In order from left to right, the pulley is assembled as such, M5 nut (P8), GT2 dual bearing idle pully (P5), precision shim (P6), then the spacer (P7), with a M5 x 25 screw (P9) through the entire assembly and corner caster bracket (P4). See Fig. 1 enlarged section. It is important to note that the shim sits in between the pulley and the spacer. The pulley assemblies (2x) can then be attached to the front rail as depicted, by attaching the cast corner bracket 2020 (P4) with a button head screw (P10) and a t-nut (P11).

**Fig. 2:**
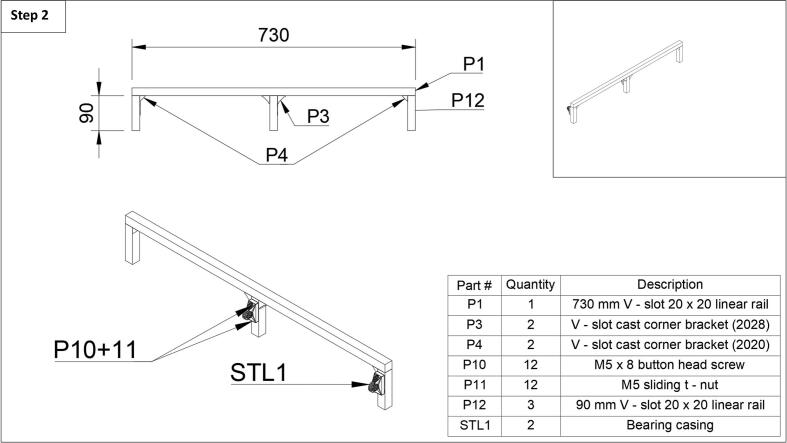
Step 2 of the fraction collector assembly guide.

Step 2: Next, the rear frame of the fraction collector and the bearing casings can be assembled. Like in step 1, start with the attachment of the center leg first. Attach two cast corner brackets 2028 (P2) to one of the three 90 mm long legs (P12) using a button head screw (P10) and t-nut (P11). Then slide two t-nuts (P11) down the rail (P1) and complete the attachment of the center, rear leg (P12) using two button head screws (P10). Next, attach the two outermost legs in a similar fashion, using button head screws (P10) and t-nuts (P11), with the smaller cast corner brackets 2020 (P4) facing inward, and the edge of the legs flush with the end of the rear rail (P1) as depicted in Fig. 2. It is important to use the smaller cast corner brackets (P4) here so that the belt will not scrape once the build is completed. Lastly, attach one bearing casing (STL1) to the center leg using two button head screws (P10) and two t-nuts (P11). Attach the last bearing casing in an identical fashion, to the leg which will be the back left leg of the fraction collector (see Fig. 2 top right). Note that the rear frame assembly uses 90 mm length legs.

**Fig. 3:**
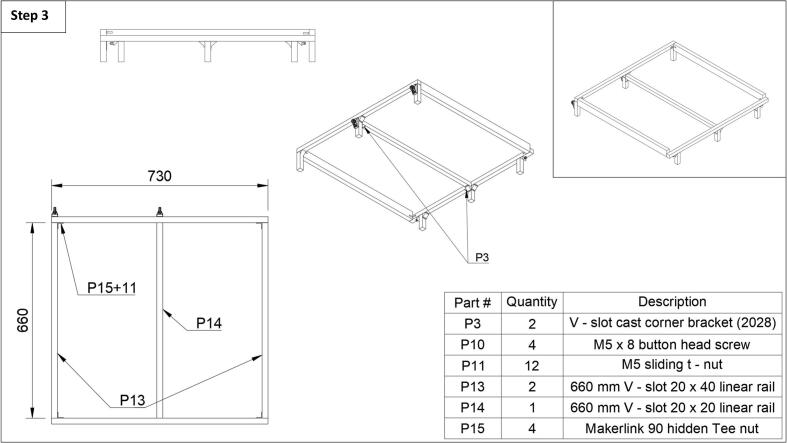
Step 3 of the fraction collector assembly guide.

Step 3: The front and rear frame can be connected using four hidden t-nut connectors and their accompanying set screws (P15) and four M5 t-nuts (P11) each, connected to the side linear rails (P13). Note that the side linear rails are 20x40 mm linear rails. The two rear hidden t-nuts (P15) are connected to the top rail slot of the side rail (P13), while the front two hidden t-nuts (P15) are connected to the bottom-most slot of the side rail (P13). See Fig. 3 for the correct assembly, it is offset. Next, the middle support rail (P14) can be attached to the center legs with cast corner brackets 2028 (P3) with two button head screws (P10) and two sliding t-nuts (P11) each.

**Fig. 4:**
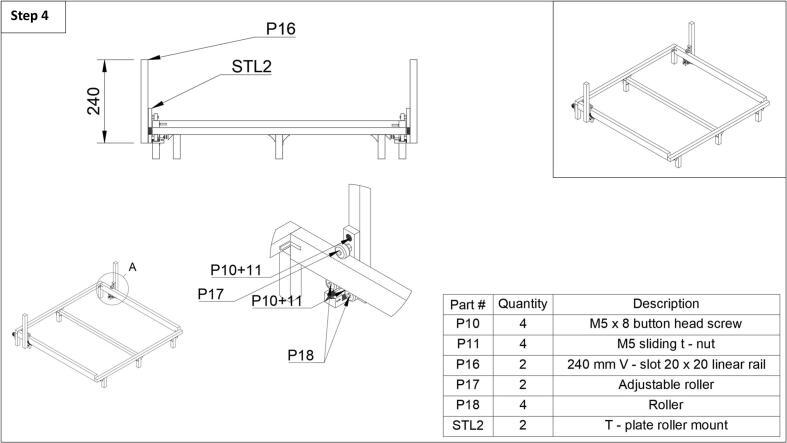
Step 4 of the fraction collector assembly guide.

Step 4: Next, each side arm can be attached. First, the adjustable (P17) and nonadjustable (P18) roller assemblies (P18) which include the roller, bearing, spacer, screw, and bolt, can be salvaged from the 3D printer. With the t-plate roller mount (STL2) placed next to the side 20x40 mm rails as shown in Fig. 4, the rollers (p17, P18) can be screwed into the plate. With the t–plate, and rollers mounted securely on the side rails, the side arm 240 mm V–slot 20x20 mm linear rails (P16) can be mounted to the t-plate using two M5 button head screws (P10) and two M5 sliding t-nuts (P11) each. For more detailed images in this section please see the extra images in the repository under the ‘step 4’ folder.

**Fig. 5:**
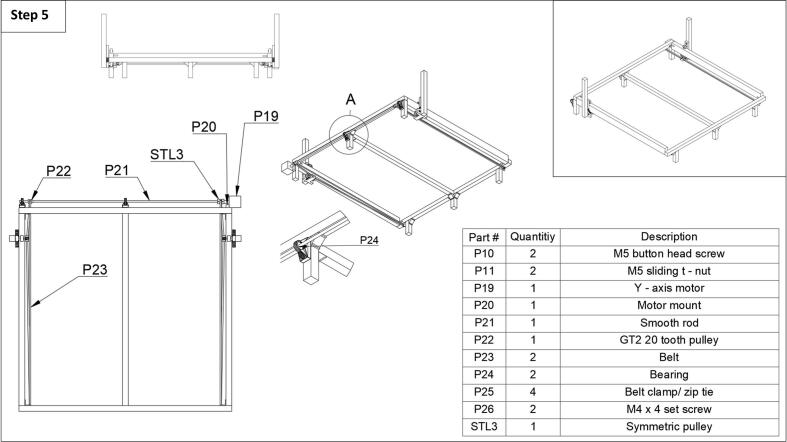
Step 5 of the fraction collector assembly guide.

Step 5: Now the rear rod, pulley, and y-axis motor can be installed. First, procure two bearings from a spare roller from the 3D printer. One roller contains two bearings, these can be obtained by cutting apart the roller (see Fig. 4**, P18**). Insert the bearings (P24) into the bearing casings. Next, slide the rod (P21) through the bearings starting from the right side of the fraction collector. Before sliding the rod through the last bearing, insert the GT2 20 tooth pulley (P22) on the rod and tighten. This pulley will connect the belt to the front left pulley of the fraction collect, ensuring they are in line with one another. Next, install the motor mount (P20) on the back right leg of the fraction collector using two button head screws (P10) and sliding t-nuts (P11). The motor mount comes from the 3D printer and is originally located connected to the z-axis motor. Next, attach the y-axis motor (P19) to the motor mount using the same screws that connected the z-axis motor to the mount on the printer. Sliding the rod back to make room, attach the symmetric 20-tooth pulley (STL3) to the rod and the y-axis motor shaft. Tighten with M4x4 set screws (P26). Then, attach the belt to the side t–plate roller mounts, around the tooth and front pulley on each side (see Fig. 5) and secure them with a belt lamp or zip tie (P25). Ensure the belts have good tension, so they do not slip on the pulley when moving, and that each side arm is in line with the other. For more detailed images in this section please see the extra images in the repository under the ‘step 5’ folder.

**Fig. 6:**
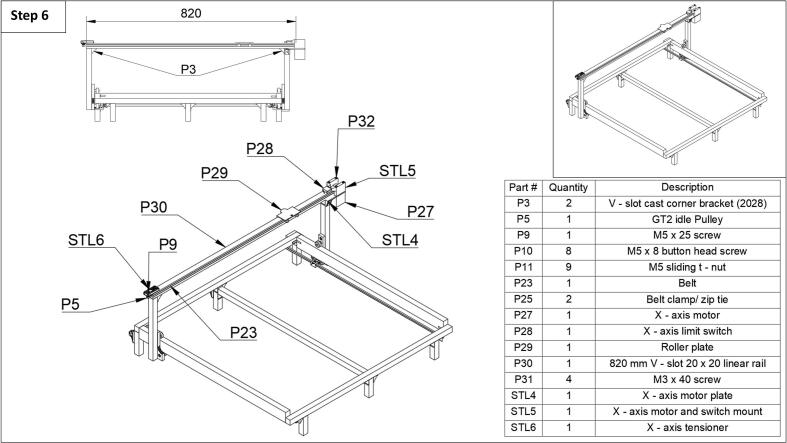
Step 6 of the fraction collector assembly guide.

Step 6: The top arm and motor assembly can be attached next (Fig. 6). First, attach the top linear rail (P30) using two corner brackets (P3), four button head screws (P10), and four sliding t-nuts (P11). Then, the motor plate (STL4) can be attached to the right vertical rail using two button head screws (P10) and two sliding t-nuts (P11). Next, the x-axis motor and switch mount (STL5) can be placed on top of the motor plate, then the x-axis motor (P27) can be placed underneath, and the whole assembly screwed together using the motor screws (P32) that came from the 3D printer (located underneath the QR code sticker, which originally held the motor in place). Following this, the x-axis limit switch (P28) can be screwed to the mount (STL5) using the same mounting screws used on the printer. Next, the roller plate (P29) which is salvaged from the printer can be installed on the opposite end of the motor mount and rolled toward the center of the assembly. Next, the X-axis tensioner (STL6) can be installed using two button head screws (P10) and two sliding t-nuts (P11). Do not fully tighten until the belt is installed to apply tension. Then, the GT2 idle pulley (P5) can be attached to the tensioner using an M5x25 screw (P31) and an additional sliding t-nut (P11), with the t-nut on the top of the tensioner. Lastly, install the belt (P23) clamped to the roller plate 29 using zip ties or belt clamps (P25). The belt will fit within the channel of the top rail. For more detailed images in this section please see the extra images in the repository under the ‘step 6’ folder.

**Fig. 7:**
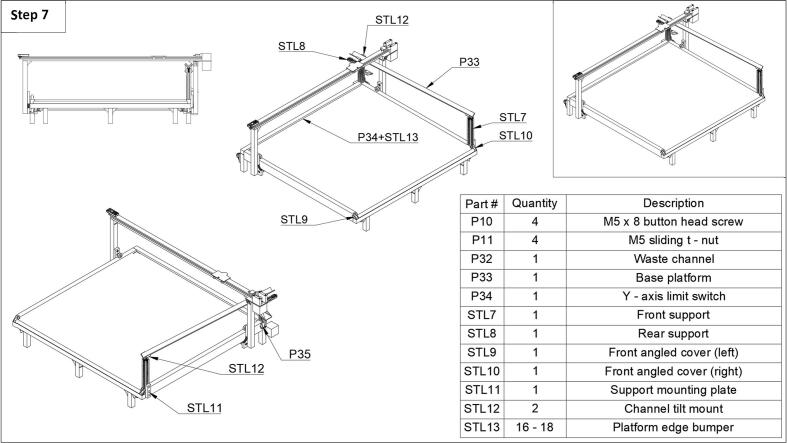
Step 7 of the fraction collector assembly guide.

Step 7: In the final step, the waste channel and base plate can be installed (Fig. 7). First, install the rear support (STL8) using one button head screw (P10) and sliding t-nut (P11). Next, install the y-axis limit switch (P35) to the rear support using the screws that came from the limit switch on the printer. Then, attach the front support (STL7) using the support mounting plate (STL11) and three button head screws (P10), and three sliding t-nuts (P11). Then install the channel tilt mounts (STL12) to each front and rear support and slide the waste channel (P33) into the mounts. The waste channel is a 90° angle stock cut to the desired length. Lastly, the base platform (P34) can be added, which was a 14 gauge/2 mm thick sheet of metal cut to size. It is recommended to measure the size base you need after the assembly of the frame but is roughly 700 mm x 670 mm. This can be slotted into the channels of the side and back rails. Then, the platform edge bumpers (STL13) can be added around the edge of the base to securely pinch the base plate down and keep the bottles away from the moving components of the fraction collector. Four corner bumpers were also designed to provide a more seamless fit (STL14). Optionally, two front corner covers can be added to reduce sharp edges (STL9 & STL10). For more detailed images in this section please see the extra images in the repository under the ‘step 7’ folder.

**Fig. 8:**
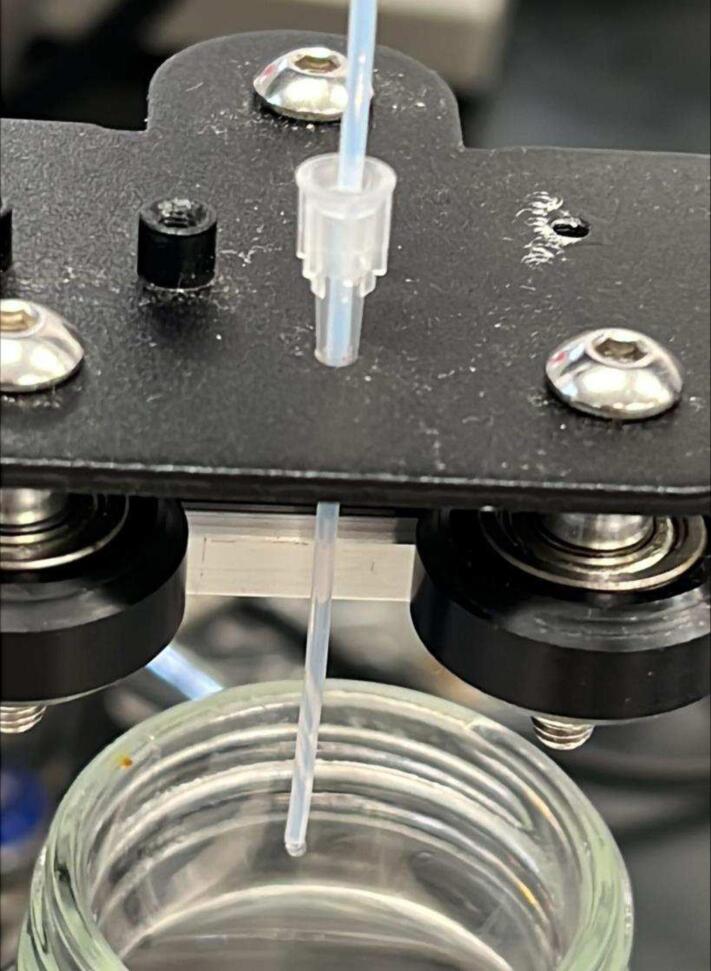
An up-close view of the roller plate (P29) with the HPLC outlet tube fit within a 10 µL pipet tip.

Step 8: Additional steps not shown in the diagram are listed here. Depending on the outlet tube of the HPLC paired to this fraction collector, it can be mounted to the roller plate (P29) in various ways. Ours was mounted using a pipet tip that was cut to allow the outlet tubing to fit snugly within (Fig. 8). It also serves to keep the line straight. With everything assembled, it is time to plug it in. The power supply (PSU), motherboard, and LCD screen can be taken from the printer and the motor and limit switch cables can then be attached, ensuring that the cables are connected correctly (x – motor cable to x-axis motor, etc.). The cables of the Ender 3 Pro come labeled. Provided in the repository are additional parts that keep the cable from being tangled and hold the modules in place, but these are secondary to the primary function of the collector. These hold the PSU, controller, and LCD screen, as well as provide a cable chain to keep the cable from becoming entangled with the moving gantry of the printer (STL15 − STL18). The chain lock has a hole which can be screwed where desired to the base but is not necessary. The chain links keep the cables from becoming tangled. Lastly, the rear support (STL11) includes an arm capable of holding a funnel, to route the waste line. Please see the extra assembly photos in the repository for completed pictures of the fraction collector.

**Flashing Firmware**.

**Fig. 9:**
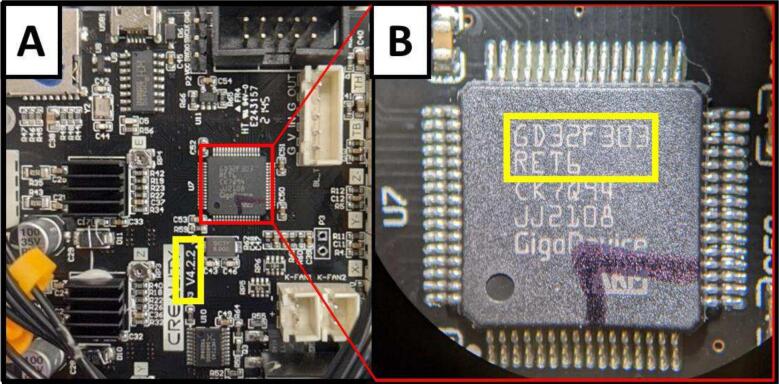
A picture of the mainboard on the Ender 3 Pro (A) with a yellow box highlighting the version number “V4.2.2” and a zoomed-in view of the chipset highlighting its model number “GD32F303RET6” (B). (For interpretation of the references to colour in this figure legend, the reader is referred to the web version of this article.)

The firmware is flashed solely to increase the x and y motor limits to accommodate the dimensions of the fraction collector. Depending on when the Ender 3 Pro was manufactured, it can come with two different motherboards or microcontroller chips. The current version of marlin firmware (v 2.1) at the time of building this robot was downloaded (Download | Marlin Firmware (marlinfw.org)), in addition to Microsoft Visual Studio Code (VSC), and the VSC plug-in “Platform.io” for free. Platform.io in VSC was used to reconfigure the maximum bed size of the 3D printer to X  = 680 mm, and Y = 540 mm. Two “.bin” files have been provided to reconfigure the firmware to increase the bed size. Files for the V4.2.2 GD32F303RET6 combination and V4.2.7 STM32F103RET6 combinations can be found in the repository in the ‘code’ folder, named with the respective board versions. This is dependent on the version of the motherboard and microcontroller chipset, which can be read on the motherboard itself (Fig. 9).

To flash the firmware, copy the “.bin” to a completely empty microSD card, ensuring it is the only file on the card and not in any folder, then insert the card into the printer. Turn the printer on and the firmware will flash, reading the.bin file automatically. This may take a few seconds to a minute. Once the printer has booted with the new firmware, the printer can be turned off and the microSD card removed. The.bin file should now be removed from the microSD and is no longer required. Turn the printer back on and use the manual control settings to verify that the bed size has been increased by setting the position of both axes past 235 mm.

This process was done following a guide on YouTube (ENDER 3 / ENDER 3 PRO With Board 4.2.2 − How To UPDATE FIRMWARE − YouTube). A similar approach can be taken if one does not have the same version/chipset, or if one decides to try to use a printer other than the Ender 3 pro for this build. Note that if a different printer is used, the salvaged parts may not be compatible with the provided.stl files or frame design and various parts in the part list may not be present.

## 6 Operation instructions

**Fig. 10:**
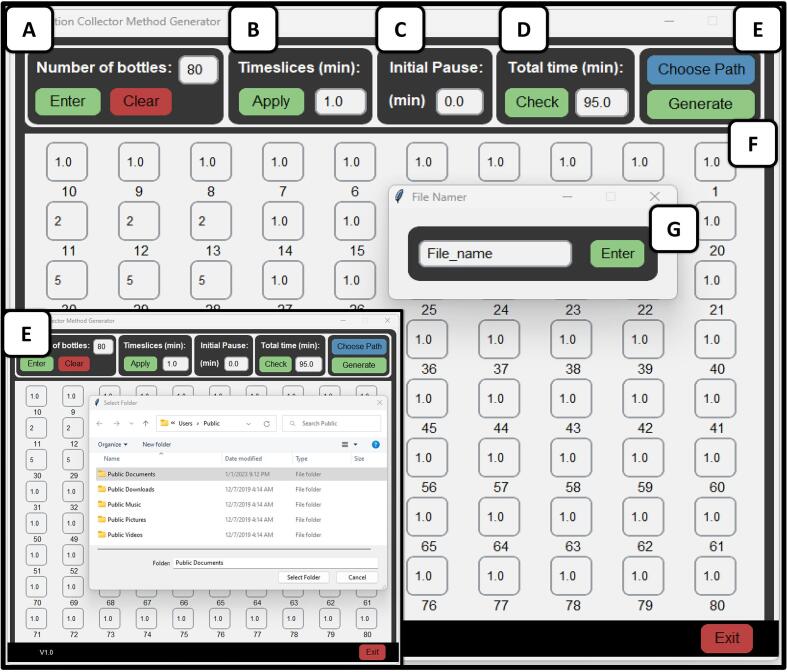
The accompanying graphical user interface allows for the number of bottle selections (A) up to 80, and how long the robot will pause on each bottle (B). An additional pause at the start of the run can be selected (C), and the total time input is added (D). A file path dialog will open upon choosing the path (E) and the method will be generated (F) after inputting the desired filename (G).

Now that the fraction collector is fully assembled, a method can be written for collection of fractions. The fraction collector operates on gcode running on Marlin firmware. A method can be made using the accompanying graphical user interface shown in Fig. 10. This is not a required software for operation. The user can also write their own method command lines with a basic text editor and save the file as a ‘.gcode’ extension.

**Method Generation (GUI)**.

The accompanying graphical user interface (GUI) was written in Python using tkinter packages. This GUI is run on a separate computer and the method is saved to the microSD card which can be transferred to the fraction collector. Abbreviated FracC for short, it is an executable application that requires two dependent.csv files for its function. These files are positional indices for the bottle position in millimeters and the position of the virtual bottle labels within the GUI. For more details on this section and examples for customization, please see the supplemental files in the repository. The repository contains a GUI folder which contains the executable and dependent positional files. Keeping these files in the same directory is required for the function of the GUI. If the user does not want to use the GUI, the provided code can also be run in Python (FracC.py file) shell or Jupyter Notebook (FracC.ipynb).

It was created so that a user of the fraction collector could generate a desired method without any knowledge of the code. Alternatively, the GUI does not need to be used to successfully operate this fraction collector, and the line-by-line gcode sequence can be written in a simple text editor such as Windows Notepad.

To operate the FracC GUI, open the executable and input the number of bottles required for the method (Fig. 10**A**). Once the number of bottles desired for the run is inputted, the “Enter” button below it can be clicked, and the window will be populated with the number of bottles inputted. From here, the time that the method pauses on each bottle for collecting the HPLC eluent can be inputted directly into each bottle, or the time can be inputted in the timeslices entry (Fig. 10**B**) and the “Apply” button can be clicked. This will update every single bottle with the inputted “Timeslice” to provide the user with a quicker method of entry should there be a conserved time in the method. If the user desires the fraction collector to pause over the waste funnel for a certain amount of time before beginning collection, this time can be entered in the “Initial Pause” section (Fig. 10**C**). Otherwise, this can be left untouched as an entry of 0 min/seconds. Once the number of bottles and timeslices for each bottle is entered, the cumulative time can be checked by clicking the “Check” button under the “Total Time” section (Fig. 10**D**). Afterward, the path in which to save the file can be selected by clicking “Choose Path” (Fig. 10**E**) which opens a file dialog. Then the method can be named and generated by clicking “Generate” (Fig. 10**F**) which opens a window in which to name the file. Once a name is typed in the new window and the enter button is clicked (Fig. 10**G**), the file will be saved. An accompanying screen recording showing the operation of the fraction collection GUI is available in the repository.

**Fig. 11:**
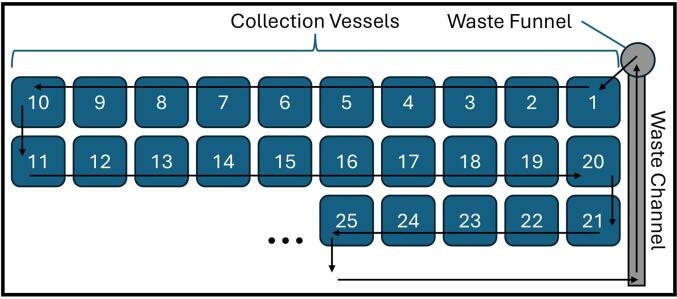
An example of the pathing of a collection method ending on the 25th bottle position. The nozzle will move from the waste funnel to bottle position one upon starting a method. To avoid adding solvent to already collected fractions 21 through 25, the path will advance one row and move directly to the waste channel, the waste funnel.

The method begins in the bottle in the top right corner next to the waste funnel and will path in a snake pattern to the left (Fig. 11**)**. Upon completion of a method the nozzle with path in a manner to avoid deposition of liquid in a previous collection vessel by advancing one row (Fig. 11), depending on bottle location. The implementation of the waste channel and collection funnel at the home position is crucial for iterative HPLC injections, since stopping the flow after every method is not typically desired. Column equilibrium will be maintained as the eluent flows to waste prior to the operator starting a subsequent injection and collecting the eluate. This end-of-sequence homing path sequence can be modified in the provided code. If the flow of the HPLC is turned off after the run, this is inconsequential.

**Running a Collection Method**.

**Fig. 12:**
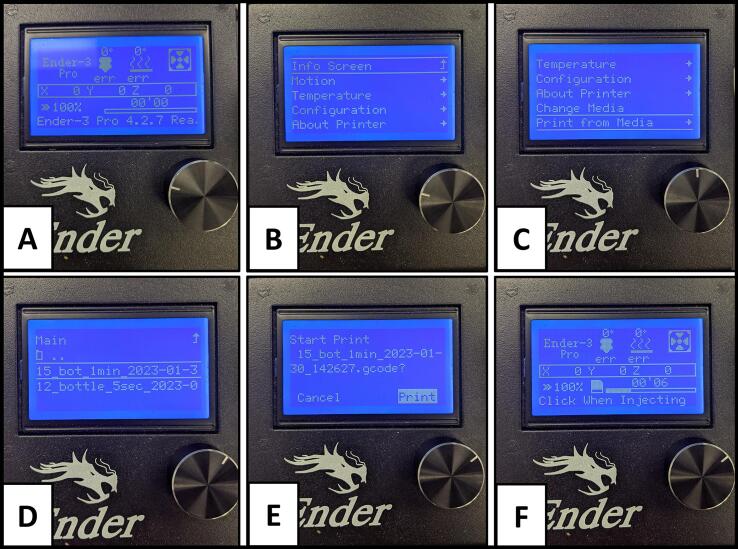
Step by step screenshots (A-F) of the Ender 3 Pro control screen when navigating to select a file to execute.

After a method has been written and saved as a “.gcode” file, it can be loaded directly on the printer using the microSD card. Alternatively, you can connect your printer directly to a computer if it has the option. First, ensure you have the appropriate number of bottles placed to collect your fractions. If leaving the flow continuously running, ensure you place extra “waste catching bottles” in the homing path (see Fig. 11 for homing path example). If the flow is stopped after the HPLC method, this can be disregarded. Next, with the HPLC equilibrated and ready for sample injection, turn on the printer with the microSD loaded. The screen should appear as Fig. 12**A**.

Click the control knob and the screen will change to Fig. 12**B**. Use the knob to scroll to “Print from Media” (Fig. 12**C**). Click the button and the screen will show the files contained in the microSD card (Fig. 12**D**). Scroll to your method using the knob and click on the method to select. A dialog will open asking if you want to print your method and will show the name of the method (Fig. 12**E).** Ensure this is the proper name of your method then use the knob to scroll to highlight “Print” and click the knob to select. This will begin the method, homing the nozzle. After the nozzle has homed, the method will pause, and a dialog will appear telling the user to “Click When Injecting” (Fig. 12**F**). At this stage, the user should load their HPLC sample and click the button at the same time their sample is injected. For details on the customization of the GUI and examples for creating methods with different bottle sizes, please see the supplemental files in the repository. It is important to note that the time it takes to move between bottles plays a role in the alignment of the chromatogram to the collected vessels. This is accounted for in the current configuration and further addressed in the supplementary customization guide in the repository.

**Synchronized Method Start**.

An entirely optional solution for synchronizing the start of the fraction collection method to the injection of a sample on the HPLC is provided. Because this increases build complexity and is only applicable to the specific use case of a manual HPLC injector, it is not considered as part of the main fraction collector building process. Synchronization increases the cost of the build (around $10 − $20 USD in parts) and complexity. The detailed parts list (Table S1) and instructions are provided in the supplementary information which can be found in the repository.

Briefly, a splitter is used to easily obtain the trigger wire from the HPLC injection port (Figure S11). A monostable − like circuit made with a 555 timer integrated circuit coupled to a relay is used to detect the voltage drop in the signal wire of the HPLC injection port, and then trigger a single button press on fraction collector, starting the fraction collection method (Figure S12**)**. This circuit can be powered using a 9 V battery for testing but can operate from 5 V – 12 V, which could be pulled from the Ender 3 Pro board. The circuit is completed by wiring the positive and negative wires from the relay to the button and soldering the wires in place (Figure S13).

## 7 Validation and characterization

**Fig. 13:**
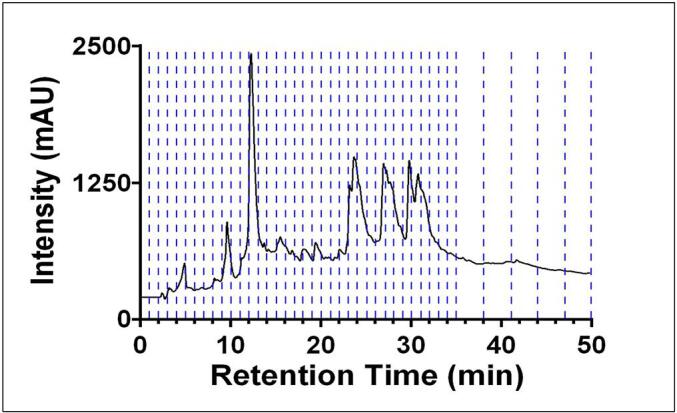
A preparative HPLC chromatogram of a complex plant extract with the corresponding time slices overlayed as collected on the fraction collector. Time slices of this method ranged from 1.0 – 3.0 min.

Although the fraction collector can be customized for a wide variety of collection methods, our initial use was intended for repeated runs of large-scale preparative HPLC, taking full advantage of the many numbers of bottles and their large volume size. Repetitive runs can be completed with each time slice corresponding to the segments of the chromatographic run (Fig. 13). This accommodates high flow rates, and repetitive collection for the subsequent concentration of eluate without the removal of collection vessels.

In this particular use case, 75.0 mg were injected in a volume of 1.0 mL on an Agilent XDB-C18 30 x 250 mm, 5 µm particle size column at a flow rate of 42.5 mL min^−1^. Multiple fractions are of interest in this sample and therefore all fractions were kept, the main fraction of interest had a 9.61 % recovery.

Under these conditions the main volume collected in a respective fraction was 42.5 mL, allowing for up to 10 runs to be repeated without having to exchange a collection vessel. Tail end collection vessels (5 fractions) had to be replaced every 3 runs but were later discarded as waste. In total, 261.4 mg of the desired fraction was recovered, an adequate amount for analytical characterization, *in vitro* and *in vivo* bioassays, and future medicinal chemistry experiments.

It is important to know any change in the tubing introduced to an LC system when paired to the fraction collector, such as the volume of the tubing post detector. This is important for aligning the peaks of a recorded chromatogram with the appropriate fraction collection bottles. For our instrument, this is 0.77 mL. At high preparatory flow rates typically ran of 42.5 mL min^−1^ shown above, this results in a maximum of 0.018 min shift from the chromatogram signal to eluate collection. This can be accounted for in the “initial pause” when creating a method (Fig. 10**D**).

At the current speed and acceleration limits set within the methods, the nozzle can move between the large collection vessels in 0.19 s. While the fraction collector was built for large vessels, the accuracy of the stepper motors from the Ender 3 Pro is as low as 0.05 – 0.1 mm, enabling smaller bottles or plates to be used.

## CRediT authorship contribution statement

**William J. Crandall:** Writing – review & editing, Writing – original draft, Visualization, Methodology, Conceptualization. **Marco Caputo:** Methodology, Conceptualization. **Lewis Marquez:** Writing – review & editing, Validation. **Zachery R. Jarrell:** Methodology, Conceptualization. **Cassandra L. Quave:** Writing – review & editing, Project administration, Funding acquisition, Conceptualization.

## Declaration of competing interest

The authors declare that they have no known competing financial interests or personal relationships that could have appeared to influence the work reported in this paper.
