# Supplementary material for: Customizable large-scale HPLC fraction collection using low-cost 3D printing
Source: HardwareX. 2024 Dec 10;21:e00612. doi: 10.1016/j.ohx.2024.e00612 (PMC11729689; doi:10.1016/j.ohx.2024.e00612)
Supplement: Supplementary Data 1 [file mmc1.docx]

Supplemental Information

Customizable Large-Scale HPLC Fraction Collection using Low-Cost 3D Printing

William J. Crandall^1^, Marco Caputo^2^, Lewis Marquez^1,3^, Zachery R. Jarrell^4^, Cassandra L. Quave^1,2^.

^1^Molecular and Systems Pharmacology Program, Emory University, Atlanta, GA, USA., ^2^Department of Dermatology, Emory University School of Medicine, Atlanta, GA, USA., ^3^Jones Center at Ichauway, Newton, GA, USA. ^4^Division of Pulmonary, Allergy, Critical Care, and Sleep Medicine, Emory University, Atlanta, Georgia 30322, USA.

# Customization

The overall process outlined in this supplemental information can be applied to different dimension builds, although if using a different 3D printer or frame material, additional 3D parts may need to be modeled.

The GUI Python code will in most cases need slight editing, so it is recommended to run this using Jupyter Notebook, as the .exe will not be able to be modified easily since it is already compiled. While the printer is designed around the position of 80 French bottles, it is possible to use other bottle types. Modification of this process in principle involves a few steps, by way of editing the dependent .csv files (“FC_Bottle_Positions_80.csv” and “Label_positions.csv”). As proof of concept, an example will be shown below under the assumption that one has 20 bottles per row, and for the sake of the exercise, a total of 100 bottles.

## Changing bottle size and GUI


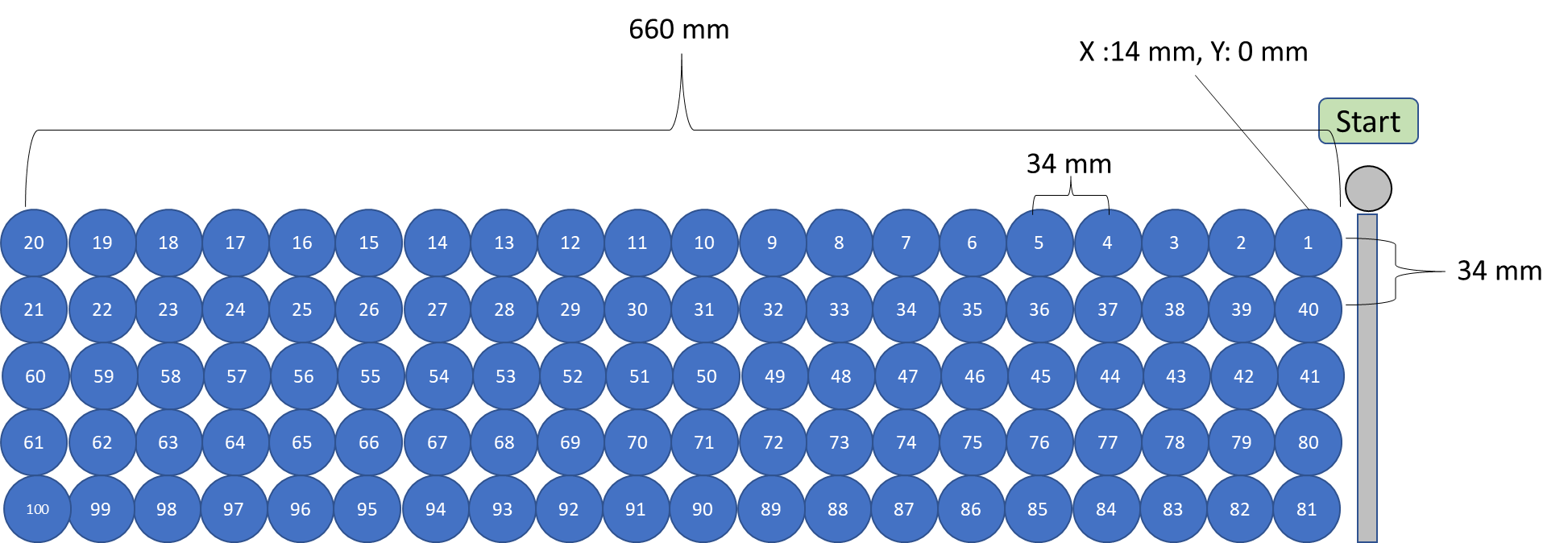


**Figure S1:** A diagram showing theoretical measurements as an example of how to customize the collector to desired bottles.

Step 1: Map the actual position of the bottles. First, place the bottles on the collector, and using the LCD screen, move the nozzle over the center of the bottles. Note the x and y coordinates (in mm) of the first bottle and the space in between bottles in both x and y directions. With smaller bottles, lining them up along the back edge might not be possible as the y = 0 position is slightly off the back line. Therefore, it is important to note the start coordinates (as shown in example **Figure S1)**. For simplicity of the example, it is assumed the starting coordinates are x: 14mm, y 0 mm. And that the distance in between the center of each bottle is 34 mm in both x and y axes.


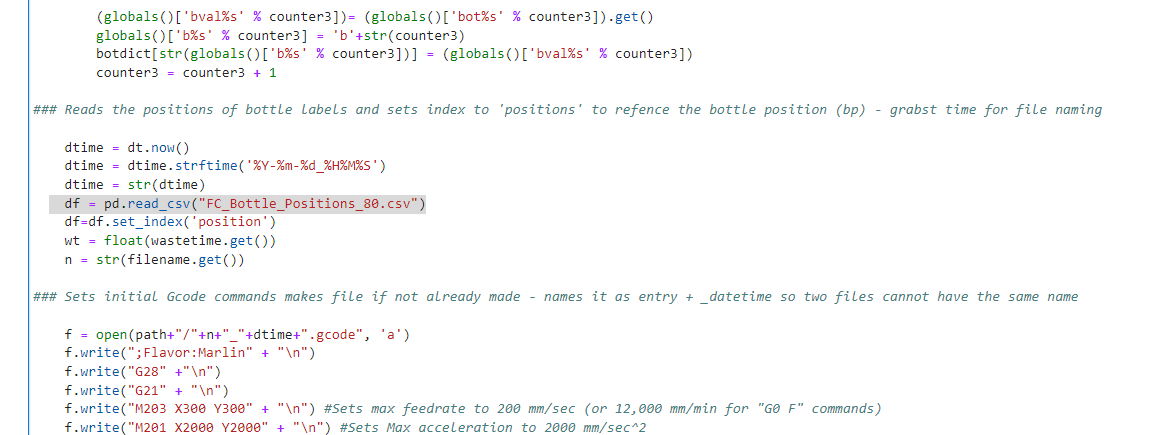


**Figure S2:** A screenshot showing the highlighted line in the FracC GUI code where the input name of the bottle positions .csv can be modified.

Step 2: Once these dimensions are known, the “FC_Bottles_Positions_80.csv” can be modified. Importantly, the name of the .csv is read by the GUI, so renaming the file will produce an error. The name of the file in the Python GUI code itself can be changed (**Figure S2)**.


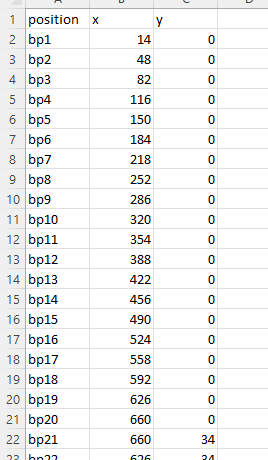
**Figure S3 (left)**: EXAMPLE - An Excel screenshot of modified “FC_Bottle_Positions_80.csv”. The mm coordinates of both x and y can be inputted for each bottle.

The position should be populated to the exact number of bottles, in this case “bp100.” With the “x” column being the x coordinate in mm and the “y” column corresponding to the y position in mm.

Step 3: Once the physical coordinates for each bottle have been inputted, this file can be saved and closed. Next, the GUI entry and text label positions must be updated to match the new number of bottles and their relative position. In cases where many bottles will be used, the size of the entry box or the window dimensions of the GUI must be changed to accommodate. In this example, the width of each entry was decreased. It will be significantly more work to change the positions of all GUI components upon resizing the window. The position should be populated to the exact number of bottles, in this case, “bp100.” With the “x” column being the x coordinate in mm and the “y” column corresponding to the y position in mm.


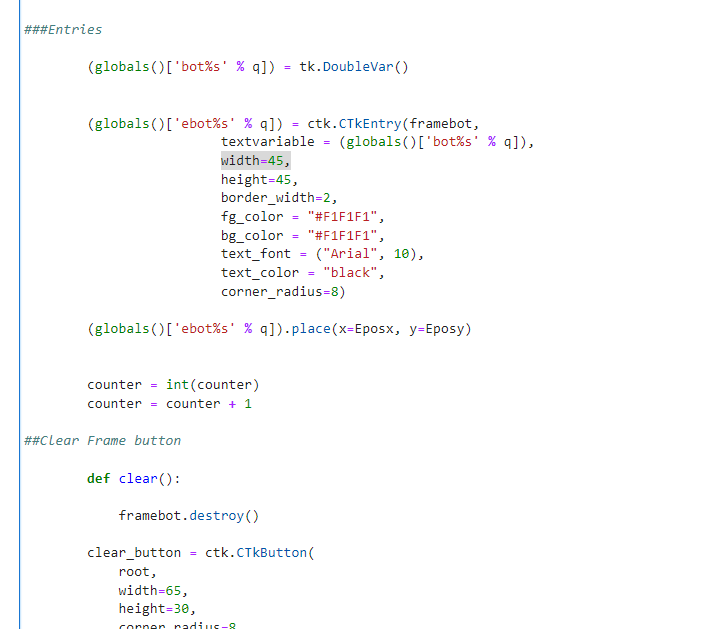
**Figure S4 (left)**: A screenshot of the highlighted line in the FracC GUI code which shows where to change the width of bottle entries.

In this example, the width was changed from 45 to 35. Afterwards, the positions of the entry (box) and the entry label (bottle number underneath) were modified in excel. Finding the correct starting coordinates, and using excel formula functions to quickly populate down the rows to create evenly spaced coordinates.


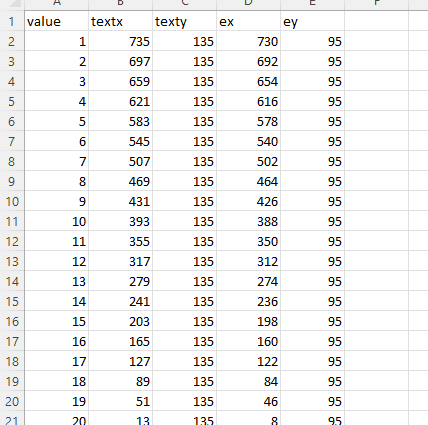


**Figure S5 (left):** An Excel screenshot showing modified coordinates for the GUI labels “Label_positions.csv”.

The value is the bottle number and should be populated to the exact number of bottles that are desired. The “textx” and “texty” columns are the x and y positions of the bottle numbers under the entries on the GUI. The “ex” and “ey” columns are the x and y positions of the entries for each bottle in the GUI. It is important to note that the end of sequence homing path was dependent on the knowledge of the number of bottles per row, and the total number of bottles. the homing path section of the script can be commented out, as shown in **Figure S6,** and an additional script written to the user’s specification. Alternatively, the provided script below will move the y-axis up 68 mm, to avoid any bottles before returning to the waste channel, and then home. If the addition of 68 mm to the y-axis exceeds 540 mm it will be reduced to not surpass this limit.

py = float(py) + 68

if py > 540:

py = 540

py = str(py)

f.write("G0 Y"+py+"\n")

f.write ("G0 F18000 X1"+"\n")

f.write ("G0 F6000 Y1"+"\n")


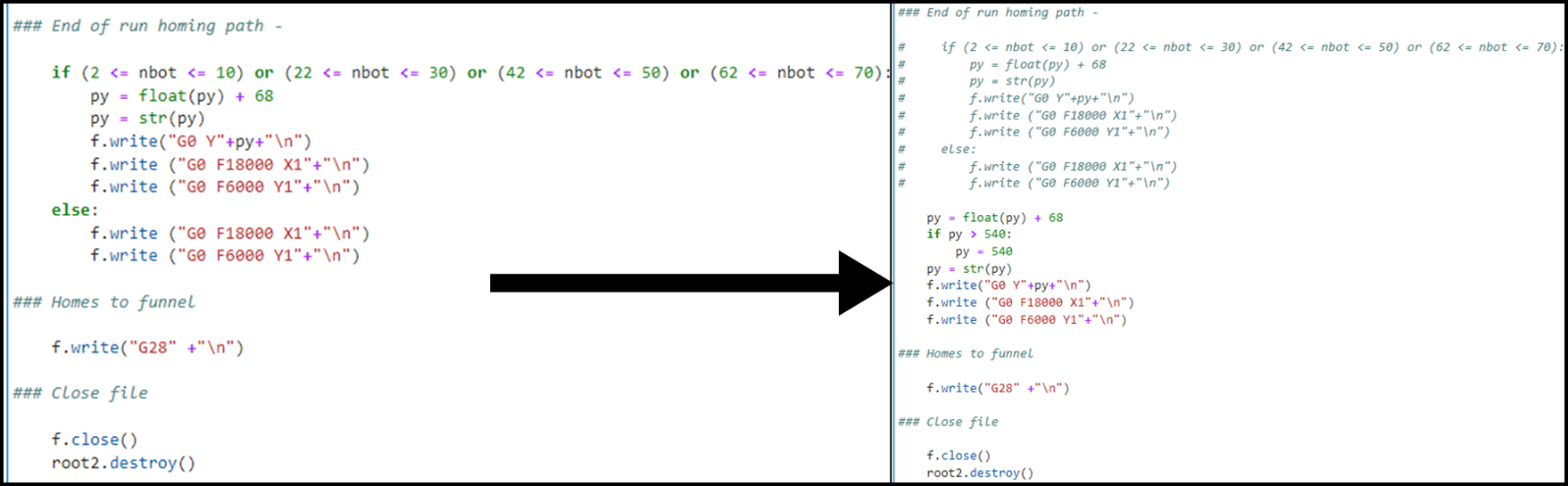


**Figure S6:** Screen shots of the FracC GUI code showing how to modify the original code (left) to accommodate different bottle layouts.

Step 3: Once these steps are completed the matching GUI (**Figure S7**) will be able to write the gcode file with the new bottle configuration. The FracC GUI can be recompiled using PyInstaller, or continued to be ran from Jupyter Notebook or another python interpreter. Note that the delay time between bottles will change and needs to be recalculated so that the chromatographic run aligns with the fraction collection method. See the “delay time” section below to complete these steps.


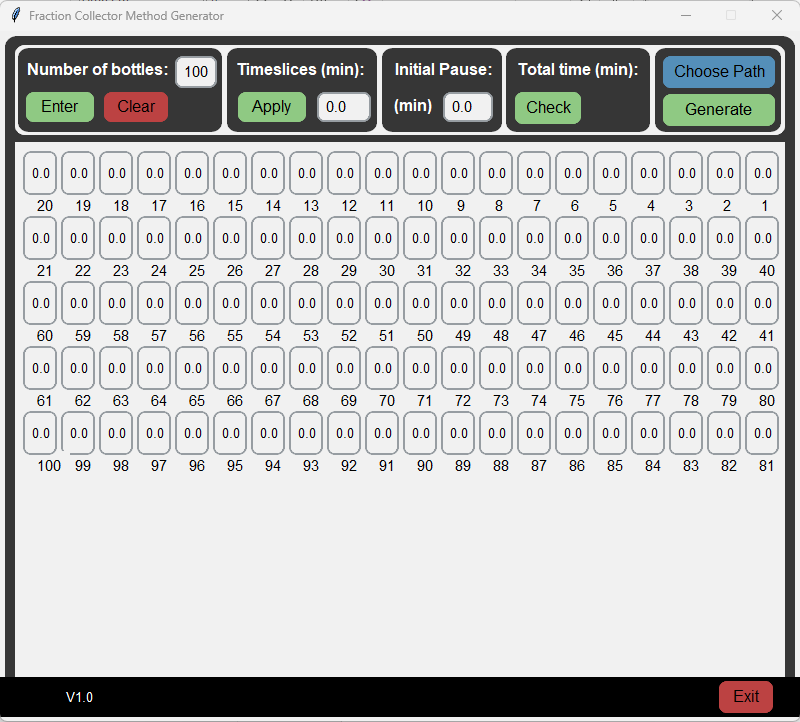


**Figure S7:** A screenshot of the new customized GUI following the example provided in this guide.

## Delay time

It is important to note that there are delays in movement time and command execution of the robot. The perceptible delay in movement time compounds throughout the duration of a run in which many movements are used. The robot was initially found to run longer than the total calculated time, resulting in the chromatographic run being finished before the robot had finished collecting fractions. To offset this, under the constant movement speed and acceleration parameters set in the .gcode files, the average delay between movements was calculated to be an average of 0.622 seconds for the large 16 oz French bottles designed for the collector. Therefore, in the code, the method is written so that every wait time command will equal the desired value with the 0.622 delays subtracted (**Figure S8)**. It is important to note that if the acceleration or movement speed of the robot is changed, this average delay will also change. This will result in mismatched chromatograms with the collected fractions. If the user is altering the GUI for different-sized bottles, this delay will also change as the movement distance will be altered. It has not been tested how this calculated delay time will affect a method for different-sized bottles, so it is recommended that the delay time be removed when creating (see **Figure S8** for the line). Once removed, a test method can be written, and the new value inputted.


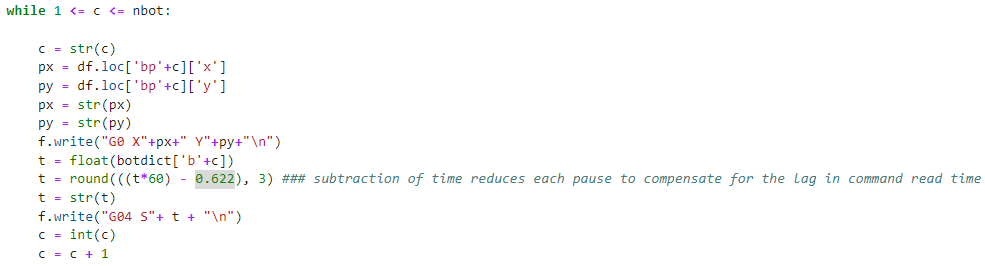


**Figure S8:** A screen shot of the GUI code, where the highlighted code shows the delay time value that needs to be subtracted from each wait time command.

For example: With 100 bottles, apply a 5-second “timeslice” or wait time for each bottle, and time how long the actual method takes the fraction collector. Theoretically, it should take 500 seconds. However, the time it takes will exceed 500 seconds due to the movement delay. This difference can be divided by the number of bottles to give the average delay time. That value can then be inputted into the code for the newly calculated delay time. If the test collection method took 560 seconds until the last fraction was collected in the method, then the total delay time was 60 seconds. Divided by 100, the delay time that needs to be subtracted from each movement is 0.60 seconds.

## Changing the Units (minutes to seconds)

The GUI is written to accommodate inputs in the unit of minutes, which must then be converted to seconds before the .gcode file is written. To change the units to seconds, simply remove the multiplicative from the code (**Figure S9**). The input of timeslices in the GUI (variable t) was multiplied by 60 to obtain the desired wait time in seconds, from which the delay time can be subtracted.


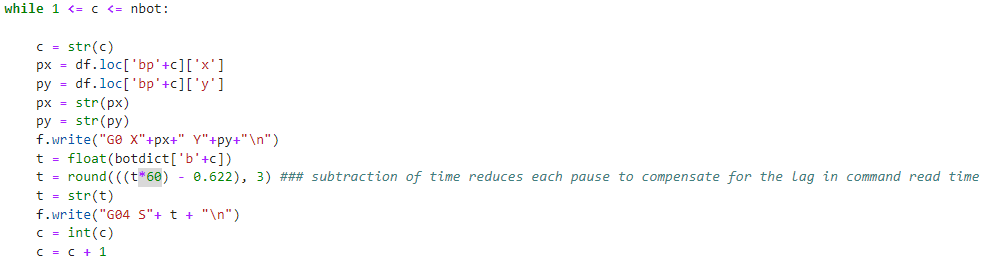


**Figure S9:** A screen shot of the GUI code with the highlighted region of the code indicating where to delete the multiplicative.

Now, any value entered in the GUI bottles will not be transformed and are therefore in units of seconds. It will also behoove the user to change the text labels in the code from (min) to (sec) which can be done by searching the code for “(min)” and altering the three places shown in red boxes in **Figure S10**.


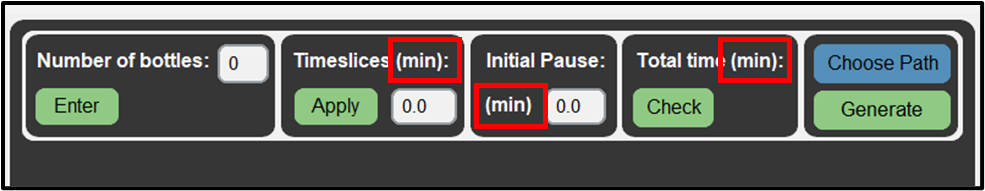


**Figure S10:** A screen shot of the GUI with red boxes highlighting the places in which the text should be changed from (min) to (sec) if altering the units of the program.

## Synchronizing the manual injector to the start of a fraction collection method

The manual HPLC injector is synchronized to the button of on the control board of the fraction collector so when the injection port is closed to the ‘inject’ position, a single signal is sent to provide a button press. This starts the method. When operating the fraction collector in this configuration it is the same as described in the paper. However, instead of clicking the button when prompted by the ‘waiting to inject’ message, the user simply must inject the sample on the HPLC normally and the method will begin automatically. The additional parts required are listed in **Table S1**.

The HPLC injector signal wire is split, and the positive lead is wired to the 555 timer integrated circuit (IC) (**Figure S11**).


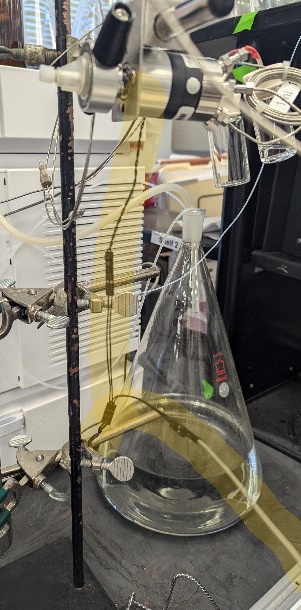
**Figure S11, left**: Splitting the signal wire with a female to 2- male connection Dupont splitter.

This is connected to the pin 2 position of the 555 timer IC according to the circuit diagram below (**Figure S12**).


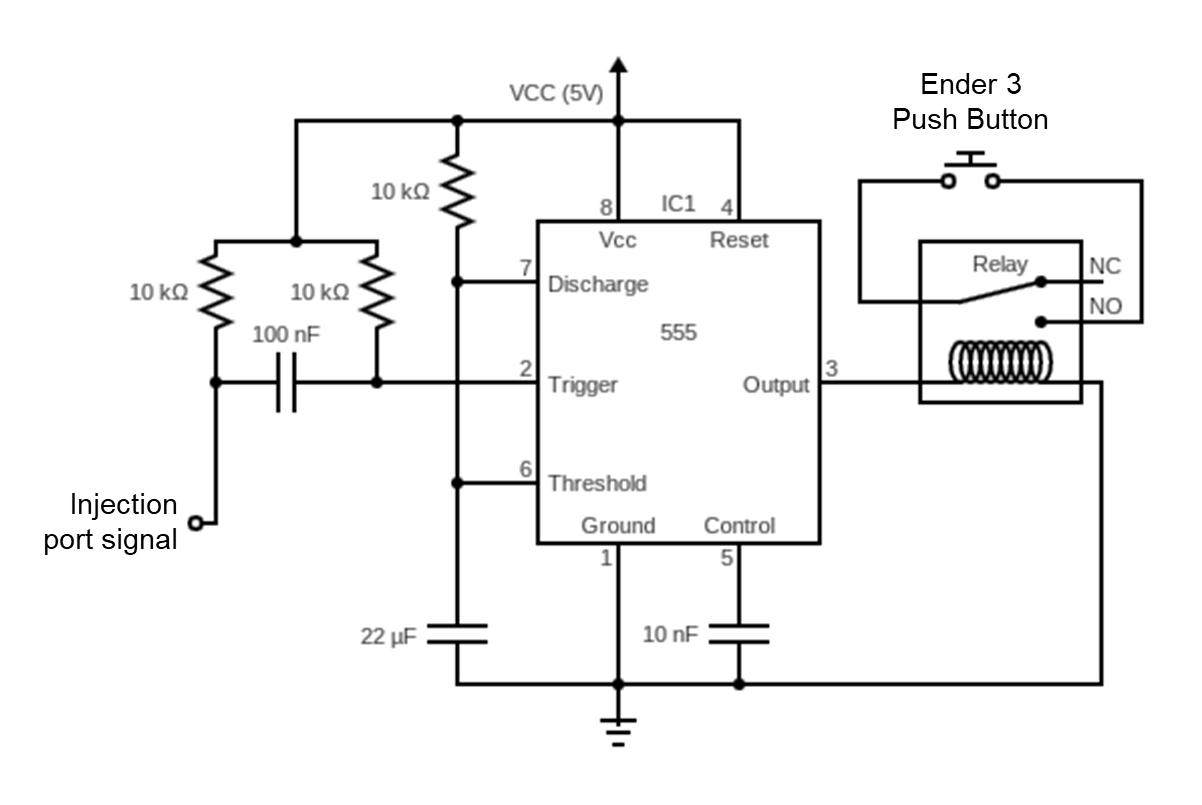


**Figure S12:** Circuit diagram for sending a single ‘button press’ signal once the injection port is set to the ‘inject’ position.

The circuit is powered by an external power source, such as a 9V battery. The output leads from the relay are then wired to the correct locations on the back of the LCD/button board on the fraction collector (**Figure S13**). It does not matter the polarity in which these are connected.


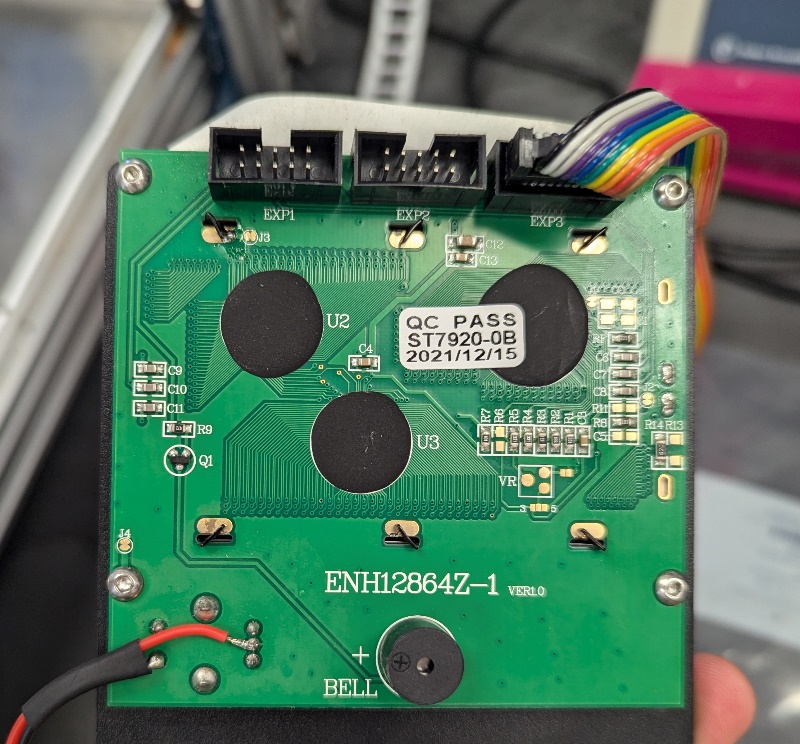
**Figure S13, left:** Positions to solder the output wires from the relay of the circuit to the ender 3 button. This input is what provides a ‘click’ to begin the fraction collection method.

**Table S1**. Extra components required for automatic method triggering.

| Component | Quantity |
| --- | --- |
| 10 nF capacitor | 1 |
| 100 nF capacitor | 1 |
| 22 uF capacitor | 1 |
| 10 kOhm resistors | 3 |
| 555 timer IC | 1 |
| 5V 5-pin relay | 1 |
| 5V -12V power supply | 1 |
| prototyping board | 1 |
| 24 gauge signal wiring as needed | ~6 ft |
| Dupont wire splitter (not required but easier to use) | 1 |
| Dupont single wire (not required but easier to connect) | 1 |
| Solder | As needed |
| 9V battery (or other power supply) | 1 |
